# Fetal Sex Modulates Hofbauer Cells’ Response to Diabetes in Human Placenta

**DOI:** 10.3390/biomedicines13112606

**Published:** 2025-10-24

**Authors:** Zdenek Tauber, Max Mrstik, Adela Burianova, Katerina Koubova, Katerina Cizkova

**Affiliations:** Department of Histology and Embryology, Faculty of Medicine and Dentistry, Palacky University, 779 00 Olomouc, Czech Republic; zdenek.tauber@upol.cz (Z.T.);

**Keywords:** Hofbauer cells, placental macrophages, diabetes mellitus, fetal sex, CD206 receptor, morphometry

## Abstract

**Background**: Hofbauer cells (HBCs) are fetal-origin macrophages in the placental villous stroma that contribute to immune tolerance at the feto–maternal interface. They predominantly display an M2 phenotype, characterized by CD206 expression. **Methods**: Using immunohistochemistry and morphometric analysis, we quantified HBCs, assessed CD206 intensity and morphology, and evaluated apoptotic body accumulation in placental villi. Comparisons were made among pregnancies complicated by type 1 diabetes mellitus (T1DM), gestational diabetes mellitus (GDM), and normoglycemic controls, as well as between male and female fetuses. **Results**: Significant effects of maternal diabetes and fetal sex on CD206 intensity were observed ([diagnosis: F = 2773.00, *p* < 0.0001; sex: F = 12.19, *p* = 0.0005]), with a strong interaction (F = 165.40, *p* < 0.0001). In controls, CD206 intensity was higher in female than male fetuses (*p* < 0.0001). Across groups, CD206 intensity decreased progressively from controls to GDM and T1DM, with a more pronounced reduction in females. Reduced CD206 was associated with elongation and irregular HBC morphology and increased IL-1β (r = −0.392, *p* = 0.003; r = −0.609, *p* < 0.0001) suggesting less tolerogenic phenotype. For apoptotic bodies, significant main effects of maternal diabetes and fetal sex were detected ([diagnosis: F = 97.16, *p* < 0.0001; sex: F = 15.88, *p* = 0.0001]). Accumulation increased progressively from controls to GDM and T1DM, with higher counts in males. **Conclusions:** Maternal diabetes is associated with reduced CD206 intensity, altered HBC morphology, and accumulation of apoptotic bodies in placental villi. Our results suggest greater HBC plasticity, potentially contributing to a tolerogenic placental environment in females.

## 1. Introduction

Successful pregnancy requires finely tuned immune regulation at the feto–maternal interface, where Hofbauer cells (HBCs), fetal macrophages residing within the placental villous stroma, play a central role in maintaining tolerance, supporting tissue remodelling, and defending against pathogens. HBCs appear in placental tissue as early as day 18 of intrauterine development and persist throughout gestation, although their numbers decline after mid-pregnancy [1]. Initially derived from the yolk sac and later from fetal monocytes [1,2,3], these cells are generally considered to exhibit an M2 macrophage phenotype, characterized by anti-inflammatory, tolerogenic, and reparative properties [4,5]. Through their involvement in villous growth, stromal remodelling, angiogenesis, and efferocytosis—the phagocytic clearance of apoptotic cells—HBCs contribute critically to placental development and immune homeostasis [2,6,7]. Their phenotype and function may be influenced by metabolic disturbances such as diabetes and by fetal sex, both of which modulate inflammatory signalling. However, despite growing evidence of these effects, no previous studies have investigated the morphological characteristics, motility, or efferocytosis efficiency of HBCs in the context of fetal sex and diabetic conditions, which is important to understanding placental immune adaptation.

CD206, also known as the mannose receptor, is one of the most widely used markers for M2 macrophages [8]. It facilitates the clearance of endogenous and exogenous molecules, contributes to antigen presentation, and regulates cellular activity to maintain tissue balance. Although classified as a scavenger receptor, its role in phagocytosis remains incompletely understood. Some studies suggest that CD206 does not directly initiate phagocytosis but rather modulates signalling through other receptors such as Fc receptors and TLRs and may also influence macrophage motility [9,10]. Macrophage motility is essential for immune surveillance, inflammation control, and tissue repair, relying on dynamic cytoskeletal remodelling and extracellular matrix interactions [11]. These mechanisms can be significantly altered under pathological conditions such as diabetes [12].

Diabetes during pregnancy is a common metabolic complication, affecting approximately 16.7% of live births worldwide [13]. It includes pre-existing type 1 (T1DM) and type 2 (T2DM) diabetes, and gestational diabetes mellitus (GDM), which accounts for about 84% of cases. Normal pregnancy involves physiological insulin resistance to optimize nutrient transfer to the fetus, whereas GDM is characterized by exaggerated insulin resistance that disrupts intrauterine conditions and promotes excessive fetal growth [14,15]. In contrast, T1DM results from autoimmune destruction of pancreatic β-cells, leading to insulin deficiency [16]. Metabolic disorders such as diabetes and obesity are linked to chronic inflammation, partly mediated by hyperglycemia-induced activation of TLR2 and TLR4 signalling [17,18]. This activation enhances cytokine release by macrophages, contributing to insulin resistance and impaired glucose metabolism [19,20]. In HBCs, a pro-inflammatory shift has been reported in T1DM [18,21], whereas such activation appears absent in GDM [4,21].

Fetal sex further influences the immune environment at the feto–maternal interface. Pregnancies with female fetuses typically exhibit a more immunoregulatory profile, while those with male fetuses show enhanced pro-inflammatory activity. These sex-related immune differences become more pronounced in pregnancy complications [22]. Notably, HBCs also display sex-specific responses, particularly under conditions such as maternal obesity and infection [23,24]. Such variations may contribute to the higher perinatal morbidity and mortality observed in male fetuses [22].

The present study aimed to investigate how diabetes (both T1DM and GDM) and fetal sex influence CD206 intensity and HBC morphology and to evaluate their potential association with inflammatory activation. In addition, this research investigates the effect of diabetes and fetal sex on the accumulation of apoptotic bodies within the placental villous stroma as a key indicator of tissue homeostasis. Moreover, we investigated the potential association between morphological characteristics, CD206 intensity and accumulation of apoptotic bodies with IL-1β that was recently published by our group [21].

## 2. Material and Methods

### 2.1. Tissue Sample Characteristics

A total of 54 formalin-fixed, paraffin-embedded samples of term placentas from healthy controls (n = 18) and patients with type 1 diabetes mellitus (T1DM, n = 22) and gestational diabetes mellitus (GDM, n = 14) were used. It is a retrospective study using archival material. The same sample set as in our previous study was used [21]. The samples were collected from 1999 to 2011 at the Department of Gynaecology, Obstetrics and Neonatology, First Faculty of Medicine and General University Hospital in Prague. Placenta samples were collected after spontaneous vaginal deliveries and cesarian sections (surgical delivery). All donors were white, Caucasian women over 18 years old who underwent routine pregnancy controls. Donor’s characteristics are summarized in Table 1; all available characteristics of the samples are provided in Supplementary File S1. The use of the samples was approved by the Ethics Committee of the University Hospital and the Faculty of Medicine and Dentistry, Palacky University, Olomouc (ref. no. 151/23).

Routine haematoxylin-eosin staining was performed on all samples to assess tissue integrity. Samples obtained from patients with T1DM and GDM showed signs of diabetic placentopathy, including edema of placenta villi and delayed maturation.

### 2.2. Immunohistochemical (IHC) Staining

Immunohistochemical detection of the target proteins was carried out on paraffin-embedded tissue sections (4 µm thick) using a two-step indirect method. Sections were first deparaffinized and rehydrated, followed by heat-mediated antigen retrieval in a citrate buffer pH 6.0. To minimize nonspecific binding, slides were treated with Protein Block (Dako, Glostrup, Denmark) for 30 min at room temperature. Primary antibody incubation was performed for 1 h at room temperature using rabbit polyclonal anti-CD206 (Abcam, Cambridge, UK, ab64693; dilution 1:1000) and mouse monoclonal anti-calreticulin (Novus Biologicals, Abington UK, NBP1-47518; dilution 1:300). Antibody dilutions were optimized based on staining intensity in positive control tissues according to manufacturer instructions, with all antibodies prepared in Dako REAL™ Antibody Diluent. Visualization of the reaction products was achieved using the Mouse/Rabbit PolyDetector DAB HRP Brown Detection System (Bio SB, Santa Barbara, CA, USA, BSB 0207). Nuclei were counterstained with hematoxylin, and Tris buffer pH 7.6 served for washing steps between reagents. Slides were subsequently dehydrated and mounted with coverslips. Each staining run included both positive and negative controls; for negative controls, the primary antibody step was replaced with Tris buffer prior to application of the detection system.

### 2.3. Image Analysis

All tissue samples were carefully evaluated by experienced histologists using ImageJ (Fiji) software ver. 1.54p. If necessary, colour deconvolution using the Colour Deconvolution2 plugin [25] was utilized. Only CD206-positive structures containing a nucleus were considered to be HBCs.

The count of HBCs was evaluated as the number of CD206+ cells per mm^2^ of villous area. In total, 4050 terminal villi were evaluated (75 villi per patient). The cell size was evaluated as the cell area in μm^2^. To describe the HBC morphology, four shape descriptors were used: circularity, aspect ratio, roundness, and solidity. Circularity is defined as (4π × area/perimeter^2^). The resulting values range from 1 to 0, where a value of 1 represents a perfect circle, and values approaching 0 indicate progressively elongated or irregular shapes. The aspect ratio characterizes the relationship between an object’s length and width, reflecting the degree of cellular elongation. It is defined as the ratio of the major to the minor axis of the best-fit ellipse fitted to each cell. Aspect ratio values are always greater than 1. Roundness is defined as [(4 × area)/(π × (major axis length)^2^]. Solidity is the ratio of the object area to the area of its smallest convex shape that contains the object (i.e., convex hull). A solidity of 1 represents a perfectly convex cell (circular or elliptical). A solidity lower than 1 indicates an irregular shape with concavities or protrusions. The more irregular or indented in shape, the lower the solidity value. CD206 intensity was measured as mean grey value after colour deconvolution and is displayed as reciprocal staining intensity in the graphs (reciprocal staining intensity (RSI) = 250 − measured staining intensity) [26]. In total, cell size, shape descriptors, and CD206 intensity were measured in 27,163 cells.

The accumulation of apoptotic bodies was measured as the calreticulin fraction in the stroma of 25 terminal villi obtained from each patient (n = 1350 in total) as follows: calreticulin fraction = (calreticulin positive area)/(area of villous stroma). Calreticulin positive area was measured after colour deconvolution. The area of the villous stroma represented the inner connective tissue without trophoblasts and the vascular area.

The obtained results were compared to our previously published data for IL-1β. The study used the same sample set stained according to the same protocol [21].

### 2.4. Statistical Analysis

The effect of diagnosis (diabetic status) and fetal sex on measured characteristics were evaluated using two-way ANOVA followed by Tukey’s post hoc test. The normality of each group was assessed using the D’Agostino–Pearson K^2^ test. The data were then log-transformed to improve the symmetry and homogeneity of variance prior to analysis. The contributions of samples’ characteristics such as mother’s age, gestation age, and method of delivery (spontaneous vs. C-section) to the obtained results were evaluated using multiple regression. Tree clustering based on shape characteristics after standardization was used. The distribution of cell clusters was analyzed using the chi-square test. To reveal the relation between the measured characteristics, Spearman correlation coefficients were calculated. All tests were performed using GraphPad Prism 8 and TIBCO Statistica software, ver. 14.0.0.15 at a level of significance of *p* < 0.05. Data are presented as median with interquartile range (IQR).

## 3. Results

### 3.1. Morphometric Analysis of CD206+ HBCs

Generally, mother’s age, gestational age, and method of delivery did not significantly contribute to the obtained results for cell count, size (area), and shape characteristics (for results, see Table 2) with one exception, namely, between method of delivery and cell size. It has been shown that the obtained differences were affected by maternal diabetes (healthy controls, GDM, T1DM) and/or fetal sex, or their interaction.

**Figure 1:**
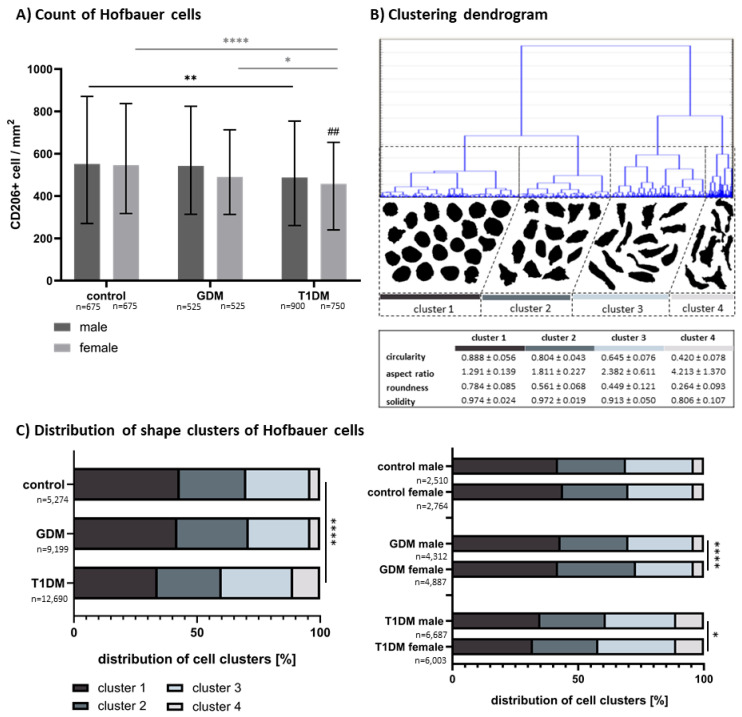
(**A**) Count of Hofbauer cells per mm^2^ of tissue. Results are displayed as median with interquartile range. The results were evaluated by two-way ANOVA [interaction: F = 2.08, *p* = 0.1250; diagnosis: F = 22.60, *p* < 0.0001; sex: F = 13.41, *p* = 0.0003] followed by Tukey’s post hoc test. Significant results within each sex are marked with an asterisk (*: black in male samples, grey in female samples) directly in the graphs: * for *p* < 0.05, ** for *p* < 0.01, and **** for *p* < 0.0001. Significant differences in female samples in comparison to male samples within the same group (control/GDM/T1DM) are marked by # directly in the graphs: ## for *p* < 0.01. (**B**) The obtained clustering dendrogram based on the shape characteristics. Using the tree joining method, four clusters were obtained. The outlines of randomly picked structures from each cluster are displayed below. Clear morphological differences are seen between the clusters. The numerical characteristics of each cluster are also displayed. (**C**) Proportions of clusters of Hofbauer cells in normal term placenta, GDM, and T1DM, as well as comparison of cluster distribution between male and female fetuses. The results were evaluated using the chi-square test. Significant results are marked by asterisks (*) directly in the graphs: * for *p* < 0.05, and **** for *p* < 0.0001.

Significant effects of maternal diabetes and fetal sex on the count of CD206+ HBCs were found (diagnosis: F = 22.60, *p* < 0.0001; fetal sex: F = 13.41, *p* = 0.0003), whereas their interaction was nonsignificant (F = 2.08, *p* = 0.1250). The number of CD206+ HBCs decreased progressively from healthy controls through GDM to T1DM. Median (IQR) counts in males were as follows: control: 551.4 (270.2–871.1), GDM: 542.5 (313.7–824.5), T1DM: 487.9 (260.7–754.0), and as follows in females: control: 546.0 (317.0–836.9), GDM: 490.5 (312.9–712.9), T1DM: 457.4 (239.9–653.6). This decreasing trend was evident in both sexes, with a more pronounced effect in females. In males, a significant reduction in CD206+ HBCs was observed between controls and T1DM (*p* = 0.0148), while other comparisons were nonsignificant (controls vs. GDM: *p* > 0.9999; GDM vs. T1DM: *p* = 0.0637). In females, T1DM differed significantly from both GDM (*p* = 0.0123) and controls (*p* < 0.0001), whereas the difference between the control and GDM groups was nonsignificant (*p* = 0.2701). Between-sex comparison within each diagnostic group revealed a significant difference only in T1DM (Tukey post hoc, *p* = 0.0043), but not in GDM (*p* = 0.1946) or controls (*p* = 0.9925). For results, see Figure 1A.

Morphological analysis of the HBCs included measurement of cell size (as cell area) and the shape characteristics, circularity, aspect ratio, roundness, and solidity. In total, 27,163 cells were analyzed. Significant effects of maternal diabetes were found for all the tested characteristics mentioned above. Significant effects of fetal sex were found for cell size, roundness, and solidity. Moreover, the interaction of maternal diabetes with fetal sex was significant for cell size, circularity, and solidity.

Based on shape characteristics (circularity, aspect ratio, roundness, and solidity), the CD206+ HBCs were divided into clusters. The clustering results based on the shape characteristics are summarized by a dendrogram in Figure 1B. Four clusters were observed. CD206+ HBCs showed a great variety of shapes. The morphological characteristics of the cells gradually change from regular and rather round with possible short protrusions (cluster 1) through triangular and oval cells (cluster 2 and 3) to elongated cells of irregular shape (cluster 4). The numerical characteristics of the shape characteristics of each cluster are also provided. The distribution of cell clusters between controls, GDM, and T1DM are summarized in Figure 1C. Distribution of cell clusters differed significantly in T1DM in comparison to control and GDM (chi-square test, *p* < 0.0001). In T1DM, the cells were shifted from cluster 1 to cluster 4. The ratio of the most elongated and irregular shapes (cluster 4) was increased to 10.8% in T1DM in comparison to 3.7% in the controls and 3.6% in GDM. Although there were significant differences in cell cluster distribution between male and female fetuses in GDM (*p* < 0.0001) and T1DM (*p* = 0.0183), there was no difference in the controls (*p* = 0.4869). The distribution of cell clusters between sexes differed in GDM and T1DM. In GDM, there was a shift in distribution in favour of cluster 2 at the expense of cluster 3 in females in comparison to males. In T1DM, there was a shift in distribution in favour of cluster 4 at the expense of cluster 1 in females in comparison to males.

### 3.2. Quantitative Analysis of CD206 and Accumulation of Apoptotic Bodies in Villous Stroma

**Figure 2:**
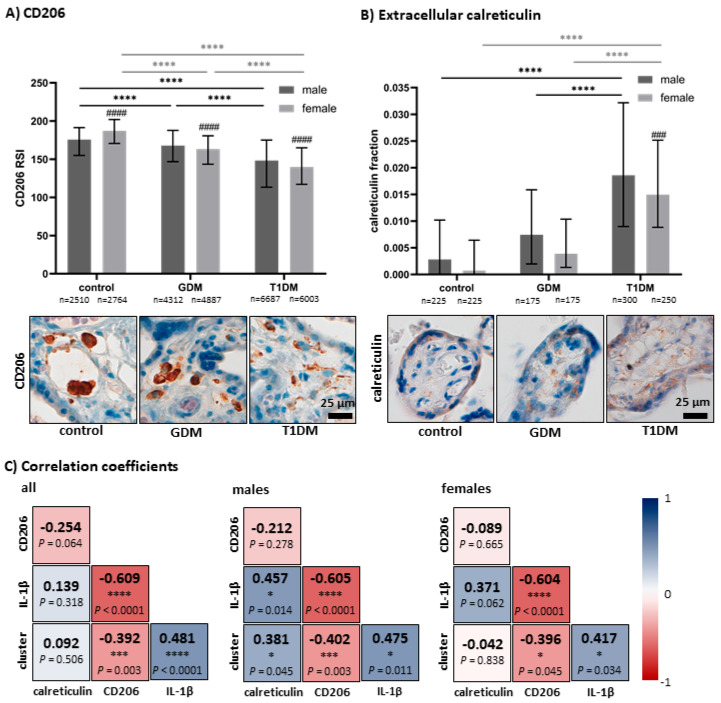
(**A**) Staining intensity of CD206, (**B**) amount of extracellular calreticulin in villous stroma with representative images. Results are displayed as median with interquartile range. The results were evaluated by two-way ANOVA [CD206: interaction: F = 165.40, *p* < 0.0001; diagnosis: F = 2773.00, *p* < 0.0001; sex: F = 12.19, *p* = 0.0005], [calreticulin: interaction: F = 2.59, *p* = 0.0755; diagnosis: F = 97.16, *p* < 0.0001; sex: F = 15.88, *p* = 0.0001] followed by Tukey’s post hoc test. Significant results within each sex are marked with an asterisk (*: black in male samples, grey in female samples) directly in the graphs: *** for *p* < 0.001 and **** for *p* < 0.0001. Significant differences in female samples in comparison to male ones within the same group (control/GDM/T1DM) are marked by a # directly in the graphs: ### for *p* < 0.001 and #### for *p* < 0.0001. All photomicrographs were obtained at a magnification of 200×; the black line represents 25 μm. (**C**) Spearman correlation coefficient demonstrating the relation between CD206, cell shape (according to cluster), extracellular calreticulin amount in villi stroma, and IL-1β. All relations were evaluated in all samples, as well as in male and female samples separately. Significant results are marked with an asterisk (*): * for *p* < 0.05, *** for *p* < 0.001 and **** for *p* < 0.0001.

Although the long-term storage of tissue samples can affect tissue antigenicity, no correlation was found between CD206 intensity and archiving time (Spearman r = 0.2190, *p* = 0.1116). A progressive decrease in CD206 intensity was observed from healthy controls through GDM to T1DM. This pattern was evident in both male and female subgroups, with a higher-fold change in females. Median (IQR) CD206 intensity values were as follows: control males: 175.7 (154.9–191.4); GDM males: 167.8 (146.8–187.8); T1DM males: 148.3 (113.4–175.0); and control females: 187.2 (170.7–202.0); GDM females: 163.3 (143.5–180.7); T1DM females: 139.8 (117.2–165.0). Significant effects of maternal diabetes and fetal sex on CD206 intensity were detected (diagnosis: F = 2773.00, *p* < 0.0001; fetal sex: F = 12.19, *p* = 0.0005), and a significant interaction between these factors was also found (F = 165.40, *p* < 0.0001). Maternal age, gestational age, and mode of delivery had no significant influence on CD206 intensity (see Table 2). Interestingly, sex-related differences were evident across all diagnostic subgroups: in controls, HBCs from female fetuses displayed higher CD206 intensity than those from males (*p* < 0.0001), whereas in both diabetic groups (GDM and T1DM), females showed significantly lower intensity than males (*p* < 0.0001 for both). For results, see Figure 2A.

Because CD206 is a receptor involved in efferocytosis, we speculated that changes in its expression may affect the clearance of apoptotic bodies. An increasing accumulation of apoptotic bodies was observed from healthy controls through GDM to T1DM. This upward trend occurred in both male and female subgroups, with a more pronounced change in males. Median (IQR) extracellular calreticulin fractions were as follows: control males: 0.0028 (0.0000–0.0102); GDM males: 0.0075 (0.0020–0.0159); T1DM males: 0.0186 (0.0090–0.0322); and control females: 0.0007 (0.0000–0.0064); GDM females: 0.0039 (0.0013–0.0104); T1DM females: 0.0150 (0.0088–0.0252). Significant effects of maternal diabetes and fetal sex were detected (diagnosis: F = 97.16, *p* < 0.0001; fetal sex: F = 15.88, *p* = 0.0001), while their interaction was nonsignificant (F = 2.59, *p* = 0.0755). When comparing male and female fetuses within each diagnostic subgroup, a significant difference was found only in T1DM (Tukey post hoc, *p* = 0.0005), but not in GDM (*p* = 0.2713) or controls (*p* = 0.9830). For results, see Figure 2B.

### 3.3. Correlation Between Measured Parameters and IL-1β

We found a significant, moderate, negative correlation between CD206 intensity and cell shape, suggesting that cells with more elongated and irregular shapes (cluster 4) showed a lower intensity of CD206. We also found a significant, strong, negative correlation between CD206 intensity and IL-1β staining intensity, suggesting inflammatory activation in HBCs with a lower intensity of CD206. Moreover, there was a moderate, positive correlation between IL-1β intensity and cell shape, suggesting higher inflammatory activation in elongated and irregular cells. These relations were found in the whole population, as well as in the male and female subgroups, at very comparable levels. Moreover, we observed the differences between male and female HBCs in terms of the relation between the number of apoptotic bodies and IL-1β staining intensity, as well as the shape of the HBCs. We found significant, moderate, positive correlations between these characteristics only in male fetuses. In females, this relation disappeared. All Spearman’s correlation coefficients are displayed in Figure 2C.

## 4. Discussion

This study investigated the impact of diabetes mellitus, specifically T1DM and GDM, on CD206 staining intensity and the morphology of HBCs to evaluate their potential association with inflammatory activation. It also explored the role of fetal sex in these parameters. Additionally, the study assessed how these factors influence the accumulation of apoptotic bodies within the placental villous stroma as an indicator of tissue homeostasis.

Since HBCs are considered M2 macrophages, we used CD206 as a marker for their identification. Our findings revealed a significant reduction in the count of CD206+ HBCs in the T1DM group, in both male and female fetuses, with a more pronounced effect observed in females. A difference in their counts between the GDM and T1DM groups was only found in female fetuses. In the current literature, there are conflicting findings regarding HBC counts in patients with diabetes. Several factors may contribute to these discrepancies. Firstly, there is currently no specific antigen for the detection of HBCs. Most studies rely on the pan-macrophage marker CD68, which may not accurately reflect the HBC population. In addition, the expression levels of many surface antigens of HBCs can vary during normal pregnancy [5] and in diabetic conditions [4,27], potentially affecting the results. Secondly, the method used to assess the counts also plays an important role. Thus, a decrease in HBCs has been reported in the T1DM group [18]. In cases of GDM, some studies have found an increase in HBCs compared to controls [28,29,30,31]. Dairi et al. have found similar counts of HBCs, or even a decrease in their levels in cases of GDM, depending on the method used [32].

In addition to the reduced number of CD206+ HBCs, our study also showed a significant decrease in CD206 intensity in HBCs from both GDM and T1DM patients compared to each other and to controls. Notably, we observed no upregulation of CD86, an activation marker of M1 macrophages, in HBCs from either diabetic group compared to those from normal placentas. These results may indicate a weakening of the M2 phenotype of HBCs in diabetic patients rather than a polarization to M1. Some authors have suggested that it is very likely that HBCs in diabetes may undergo an inter-subgroup transformation or even transform into a new, unique phenotype, rather than simply maintaining M2 polarization or transforming into the M1 phenotype, which is consistent with our results [33]. In normal female placental samples, we observed significantly higher CD206 staining intensity compared to males, which may indicate a stronger M2 phenotype. This is consistent with the findings of Paparini et al. [34]. In both types of diabetes, the reduction in CD206 intensity in the HBCs was more pronounced in females, suggesting a greater deviation from the baseline phenotype and possibly reflecting their greater plasticity. This interpretation is further supported by a study by Pantazi et al. [23], who demonstrated a stronger and more diverse HBC response to LPS stimulation in females compared to males.

It has been shown that the phenotype and cytokine production of macrophages can be related to their morphology [35]. Many authors describe round macrophages as the M2 phenotype and elongated macrophages as the M1 phenotype [36,37,38], although there are contrary opinions [35]. However, it has to be taken into account that practically all the above-mentioned papers evaluated the morphology of macrophages and their biological behaviour in vitro, using different cell lines and different activation and maturation protocols. Furthermore, some experiments on macrophages have been performed using different biomaterials. To the best of our knowledge, our study is the first to evaluate the morphology of HBCs under diabetic conditions in situ.

Generally, diabetes is associated with inflammation; GDM is considered to be a low-grade inflammatory state while T1DM is associated with a more pronounced activation. In our previous work [21], we demonstrated that significant inflammatory activation of HBCs, evidenced by an increase in IL-1β, occurs only in T1DM, and not in GDM or normal placentas. In this study, we identified a significant negative correlation between CD206 and IL-1β, a pattern consistent across both sexes and in the overall population. This finding aligns with existing literature, which reports that reduced CD206 intensity in macrophages contributes to the development of inflammation [39]. Furthermore, we observed a significant increase in irregular and elongated HBC morphology in T1DM placentas compared to controls. These morphological changes also showed a negative correlation with CD206, both in the overall population and when males and females were analyzed separately.

The CD206 antigen may be associated with macrophage motility [40]. Macrophage motility is important to facilitate their defensive response, which requires the reassembly of the actin cytoskeleton, formation of cell structures, and adhesion to the extracellular matrix [11]. Sturge et al. showed that CD206-deficient bone marrow-derived macrophages exhibit increased migration independent of a CSF-1 gradient [10]. One possible explanation is that downregulation of CD206 in macrophages is associated with the activation of RAC1/CDC42/PAK1 kinase, which plays a key role in cytoskeletal remodelling [41]. In addition, Barraza et al. showed that IL-1β activates the same kinase and is associated with cell elongation [42]. These findings are also consistent with the positive correlation between IL-1β and the irregular, elongated shape of the HBCs observed in our study.

The morphology of macrophages has been demonstrated to be associated with the type of their movement. Circular macrophages with protrusions have been observed to move in an amoeboid manner that is both faster and less persistent (i.e., the sprinter type). This mode of movement is effective for shorter-distance migration. In contrast, elongated macrophages have been shown to move in a mesenchymal manner that is slower but more persistent (i.e., the marathoner type). Thus, mesenchymal movement is considered more efficient for migration over longer distances [43]. Macrophage motility is significantly affected by the composition of the extracellular matrix (ECM). In the presence of fibronectin, cells exhibit a mesenchymal mode of movement, whereas laminin promotes an amoeboid one [44]. A significantly higher fibronectin content has been shown in diabetic placentas compared to normal placentas, whereas laminin content was not affected [12]. This environment predisposes macrophages to a mesenchymal movement. We observed more pronounced changes in the morphology of HBCs in female fetuses in diabetic pregnancies, especially in T1DM, where they were most elongated. Thus, our results suggest that HBCs in males move in an amoeboid manner more frequently, whereas mesenchymal movement plays a greater role in females. With regard to the composition of the ECM in diabetes, the morphological changes in HBCs we have shown may favour females over males. The observed differences between the sexes may be underlined by a differential arrangement of HBC cytoskeletons. Pantazi et al. observed that HBCs from females overexpressed several proteins primarily associated with the cytoskeleton, adhesion, and membrane configuration in response to LPS compared to males [23]. Variations in HBC metabolism may also contribute, as different modes of macrophage migration could require distinct energy sources. Paparini et al. (2024) demonstrated that female placental macrophages primarily utilize fatty acids, whereas male macrophages rely on glucose [34].

One of the key functions of HBCs is to establish and maintain immunological tolerance at the feto–maternal interface. In this regard, efferocytosis plays an important role, and dysregulation of this process is associated with pregnancy complications [45,46]. Our study showed that both diagnosis and fetal sex have an impact on the accumulation of apoptotic bodies in the placental villous stroma. Their levels show a progressive increase from normal pregnancies through GDM to T1DM. The existing literature on the presence of apoptotic cells in the placentas of diabetic patients remains inconclusive. Although some studies report increased apoptosis in diabetes [47,48], other studies suggest a decrease in the number of apoptotic cells compared with the normal placenta [49,50]. It is known that efferocytosis is more efficient in female macrophages than in male ones [51,52] and this has subsequently been confirmed for HBCs as well [34]. Our results also suggest that efferocytosis is more efficient in females.

In addition to sex hormones, there are a variety of factors that favour females in ensuring continuous efferocytosis, such as differences in the expression of certain receptors, particularly TLRs, the cytoskeleton, and metabolic differences [23,34,51,53]. Although HBCs are generally considered to be tolerogenic macrophages, partial depletion of CD206 has been shown to stimulate efferocytosis and is associated with the production of pro-inflammatory cytokines, which may play costimulatory and homeostatic roles [54,55]. Therefore, the more pronounced decrease in CD206 in female HBCs with T1DM may contribute to a more effective efferocytosis. This may be associated with a lower accumulation of apoptotic bodies in the stroma of the placental villi. Our results suggest that female fetuses maintain a more stable and tolerogenic environment in the diabetic placental villi compared to males. It is well known that increased accumulation of apoptotic bodies is associated with a higher risk of chronic placental inflammation and preterm birth [56]. Although we observed a greater accumulation of apoptotic bodies in males, we did not find a significant correlation between their accumulation and gestational age in our sample. Nevertheless, it is widely recognized that the incidence of preterm birth and perinatal morbidity is higher in male fetuses [22,57]. Placental inflammation may also have long-term consequences. In particular, its association with the occurrence of a spectrum of neuropsychiatric and behavioural disorders is currently the subject of intense research [58,59]. With regard to inflammation, the changes observed in GDM are minimal, whereas those in T1DM are much more pronounced. This does not mean that GDM is a negligible pregnancy complication. HBCs perform a variety of functions in the placenta, including vasculogenesis, and as such need to be considered from multiple perspectives. In GDM, our previous work has demonstrated a severe degree of hypovascularisation in the placental villi, accompanied by significant expression of soluble epoxide hydrolase in HBCs [21]. This highlights the fundamentally distinct nature of the two pathological conditions and suggests that their potential consequences for the fetus may also differ.

We acknowledge the limitations of this study, particularly those related to its retrospective design and the use of archival tissue samples. Data on the BMI of pregnant women were not available, and we lacked samples of patients with pre-existing T2DM. In addition, detailed information on treatment modalities and the level of diabetes compensation over time, which could influence the results [27], was not available. It is also known that the long-term storage of samples can affect tissue antigenicity [60]. However, in our study, no correlation was found between the length of archiving time and the CD206 intensity. On the other hand, immunohistochemical detection of antigens in situ within HBCs effectively addresses some of the limitations of in vitro studies. A major limitation of in vitro experiments on HBCs is the potential contamination of samples by maternal macrophages. In addition, macrophages are highly sensitive to their microenvironment, and it is challenging to precisely mimic diabetic conditions. Besides the microenvironment, it is well known that different populations of macrophages and tissues exhibit varying levels of efferocytosis and expression of different receptors, including CD206. This suggests that the receptors involved in efferocytosis may depend on the tissue context and the type of apoptotic cell being engulfed. Due to the complexity of this phenomenon, further research in this area is required.

Taken together, our results indicate a reduction in CD206+ HBCs under diabetic conditions compared to normal placentas. In the control group, CD206 intensity was higher in female fetuses than in males. Both T1DM and GDM were associated with decreased CD206 intensity. Furthermore, a decrease in CD206 intensity correlated with elongation and irregular morphology of HBCs. These phenomena are more pronounced in female fetuses, which deviate more from baseline than males. Besides changes in morphology, a decrease in CD206 is associated with an increase in IL-1β. In addition, we observed an increased number of apoptotic bodies in the villous stroma under diabetic conditions, with a higher incidence in male fetuses. This could potentially contribute to an increased risk of chronic inflammation and preterm birth. Collectively, these findings suggest a greater plasticity of HBCs in females, potentially contributing to a more tolerogenic placental environment.

## Figures and Tables

**Table 1:** Characteristics of patient samples.

	Control	GDM	T1DM
mother age			
mean ± SD	30.1 ± 4.2	30.4 ± 2.8	31.7 ± 5.5
median (IQR)	30.0 (26.75–31.50)	30.0 (28.0–32.25)	33.0 (28.75–36.0)
duration of diabetes			
mean ± SD	-	-	12.8 ± 6.8
median (IQR)	-	-	13.5 (6.75–17.75)
gestational age			
mean ± SD	39.1 ± 0.9	38.9 ± 1.1	37.4 ± 2.3
median (IQR)	39.0 (38.0–39.25)	39.0 (38.0–40.0)	38.0 (36.75–39.0)
fetal sex	9 males, 9 females	7 males, 7 females	12 males, 10 females

**Table 2:** Multiple regression results (n = 54), level of significance: *p* < 0.05. Significant factors are bold.

		b	std. Err. of b	*p*-Value
CD206 intensity	mother age	−1.1433	0.8400	0.1796
	gestational age	1.1224	2.0931	0.5942
	delivery	−5.3578	7.7942	0.4950
calreticulin fraction	mother age	−1.1494	0.8397	0.1772
	gestational age	1.1969	2.0924	0.5699
	delivery	−6.2947	7.7918	0.4230
cell size	mother age	−0.3228	0.4593	0.4854
	gestational age	1.3679	1.1445	0.2377
	**delivery**	**9.1660**	**4.2619**	**0.0364**
circularity	mother age	−0.0009	0.0012	0.4387
	gestational age	0.0011	0.0029	0.6960
	delivery	−0.0068	0.0107	0.5285
aspect ratio	mother age	0.0102	0.0077	0.1895
	gestational age	−0.0298	0.0192	0.1266
	delivery	0.0208	0.0714	0.7726
roundness	mother age	−0.0018	0.0015	0.2449
	gestational age	0.0065	0.0039	0.1000
	delivery	0.0022	0.0144	0.8789
solidity	mother age	−0.0002	0.0005	0.7166
	gestational age	−0.0007	0.0012	0.5531
	delivery	−0.0063	0.0044	0.1611
cell cluster	mother age	0.0054	0.0073	0.4617
	gestational age	−0.0182	0.0182	0.3222
	delivery	0.0247	0.0680	0.7180
CD206+ cells per mm^2^	mother age	0.0623	5.4150	0.9909
	gestational age	1.1433	13.4936	0.9328
	delivery	−69.5418	50.2477	0.1725

## Data Availability

Data is contained within the article or Supplementary Materials.

## Supplementary Materials

The following supporting information can be downloaded at: https://www.mdpi.com/article/10.3390/biomedicines13112606/s1, Supplementary File S1: All available characteristics of used sample set. The same sample set was used in our previous study [21]. C-section: cesarian section, F: female, M: male.
