# Supplementary material for: Fetal Sex Modulates Hofbauer Cells’ Response to Diabetes in Human Placenta
Source: Biomedicines. 2025 Oct 24;13(11):2606. doi: 10.3390/biomedicines13112606 (PMC12650028; doi:10.3390/biomedicines13112606)
Supplement: Supplementary file 1 [file biomedicines-13-02606-s001.zip › biomedicines-3930355-supplementary.pdf]

Supplementary file S1: All available characteristics of used sample set. The same sample set was used in our previous study [21]. C-section: caesarean section, F: female, M: male.

type 1 diabetes mellitus (T1DM)							
No.	mother´s age (years)	duration of diabetes (years)	HbA1c	gestation week	delivery	placenta (g)	newborn (gender/g/cm)
1	34	20	7.7	38	C-section	655	F / 3630 / 49
2	35	6	3.8	38	C-section	645	M / 3680 /52
3	33	15	3.7	40	C-section	620	M / 3790 /51
4	21	7	5.8	37	spontaneous	550	M / 2920 /47
5	36	17	4.0	39	C-section	770	F / 4070 / 53
6	23	1	3.1	39	spontaneous	490	F / 3170 /49
7	36	15	3.3	38	C-section	475	F / 2860 / 46
8	31	13	4.5	37	C-section	560	M / 3570 / 51
9	28	14	4.8	39	C-section	500	M / 3390 / 48
10	24	11	4.7	37	C-section	400	M /2400 / 42
11	38	27	5.0	38	C-section	680	F / 4290 / 52
12	42	14	4.4	36	C-section	695	M / 4200 / 55
13	31	7	3.8	40	C-section	615	F /2880 /47
14	33	10	4.8	39	spontaneous	610	M / 3430 / 49
15	38	4	5.0	35	C-section	310	F / 1845 / 41
16	30	10	4.3	40	spontaneous	560	F /3900 / 51
17	29	6	7.2	38	C-section	620	F / 4480 / 53
18	36	23	4.1	34	C-section	370	F / 2230 / 46
19	34	15	3.4	39	spontaneous	525	M / 3210 / 49
20	34	5	9.4	32	C-section	754	M / 2900 / 45
21	30	21	5.2	32	C-section	845	M / 3260 / 50
22	22	20	7.7	38	C-section	760	M / 3950 / 49

gestational diabetes (GDM)						
No.	mother´s age (years)	treatment	gestation week	delivery	placenta (g)	newborn (gender/g/cm)
23	27	diet	39	C-section	450	M / 3010 / 50
24	28	diet	38	spontaneous	570	M / 3140 / 43
25	28	-	39	C-section	1000	M / 3760 / 51
26	27	insulin	40	spontaneous	520	M / 3250 / 50
27	29	insulin	39	spontaneous	560	M / 3080 / 50
28	32	diet	41	spontaneous	585	F / 3940 / 54
29	31	diet	39	spontaneous	725	M / 3555 / 53
30	30	insulin	37	spontaneous	500	M / 3020 / 50
31	31	diet	40	spontaneous	550	F / 3840 / 50
32	34	insulin	40	spontaneous	590	F / 3280 / 50
33	37	-	39	spontaneous	570	F / 3470 / 51
34	29	-	38	spontaneous	405	F / 2910 / 47
35	30	insulin	37	spontaneous	476	F / 2570 / 46
36	33	diet	39	spontaneous	340	F / 3330 /51

controls					
No.	mother´s age (years)	gestation week	delivery	placenta (g)	newborn (gender/g/cm)
37	30	39	C-section	900	M / 3700 /
38	27	40	C-section	555	F / 3030 /
39	30	39	C-section	635	M / 3870 / 52
40	24	40	C-section	620	M / 3730 / 53
41	26	38	C-section	435	F / 2780 / 45
42	33	38	C-section	700	M / 3470 / 50
43	37	39	C-section	740	M / 4110 / 53
44	24	38	spontaneous	390	F / 2890 / 50
45	36	38	C-section	770	F / 4200 / 51
46	30	39	spontaneous	600	F / 3380 / 49
47	38	39	spontaneous	535	M / 3490 / 50
48	31	39	C-section	605	M / 3560 / 50
49	23	39	spontaneous	580	F / 3520 / 52
50	31	38	C-section	590	F / 3175 / 50
51	31	39	C-section	603	F / 3880 / 50
52	30	41	C-section	575	M / 3180 / 49
53	30	41	C-section	640	M / 3820 / 52
54	30	39	C-section	460	F / 3250 / 49
